# Women and the State

**Published:** 1920-03-06

**Authors:** 


### Women and the State.

### Quickening Interest at Home and Abroad.

The whole subject, national and international, of women's influence in the life of the State is growing in substance and interest as never before. There have been several outstanding manifestations of this during the last few days. For instance, here at home, the House of Commons has by a considerable majority voted its support of a private Franchise Bill, giving the vote to five million young women between the ages of twenty-one and thirty. Nearly all the speeches paid tribute to the new spirit which women were infusing into public life, and, as Dr. Addison himself quietly reminded the House, the sympathetic attitude towards, and almost eager support of, the Bill was in interesting contrast with the acrimony that such subjects engendered in Parliament not so many years ago.

Then there was the Conference of Women Organisations, held at Caxton Hall to consider the representation of women in the League of Nations, especially in dealing with questions of child and adolescent labour and other social questions. The discussion showed what interesting and important work women might be called upon to do, and how necessary it was that the different countries should be able to draw from a large list of women possessing the qualifications which fitted them for highly expert social work. For instance, one of the speakers said that, if an Allied Labour Commission were sent to investigate the conditions in Russia it would be necessary to have on that Commission at least one woman capable of getting into touch with Russian women of all classes, and collecting their evidence. An effort is to be made by a provisional Committee to secure a list of qualified women able to act in various capacities.

There is also a preliminary announcement of the International Council of Women, which is to meet in Christiania this year. The agenda includes such things as the consideration of forming an international bureau of education, on which women would be adequately represented, and also the question of an International Bureau of Public Health, as part of the organisation of the League of Nations. This last point is already under way, having been undertaken by the League itself, but no doubt the Conference in Christiana will have some suggestions to make. The international movement for child welfare is exemplified in a resolution calling upon women of all countries to work with energy and devotion for the welfare of children, and especially to do all in their power to save the children in the famine stricken districts of Europe. These are just a few instances of the growing international outlook of women's organisations.

